# Modulating Effects of Spirulina platensis against Tilmicosin-Induced Cardiotoxicity in Mice

**DOI:** 10.22074/cellj.2015.520

**Published:** 2015-04-08

**Authors:** Abdelaziz E. Ibrahim, Mohamed Mohamed Abdel-Daim

**Affiliations:** 1 Department of Forensic Medicine and Toxicology, Faculty of Veterinary Medicine, Suez Canal University, Ismailia, Egypt; 2 Department of Pharmacology, Faculty of Veterinary Medicine, Suez Canal University, Ismailia, Egypt

**Keywords:** Tilmicosin, Oxidative Stress, Spirulina, Antioxidant, Heart

## Abstract

**Objective:**

Tilmicosin (TIL) is a long-acting macrolide antibiotic used to treat cattle for pathogens that cause bovine respiratory disease. However, overdoses of this medication have been reported to induce cardiac damage. Our experimental objective was to evaluate the protective effects of *Spirulina platensis* (SP) administration against TIL-induced cardiotoxicity in mice.

**Materials and Methods:**

Our experimental *in vivo* animal study used 40 male albino mice that were divided into five groups of eight mice per group. The first group served as a control group and was injected with saline. The second group received SP at dose of 1000 mg/kg body weight for five days. The third group received a single dose of TIL (75 mg/kg, subcutaneously). Groups 4 and 5 were given SP at doses of 500 and 1000 mg/kg body weight for five consecutive days just before administration of TIL at the same dose and regimen used for group 3.

**Results:**

TIL treated animals showed a significant increase in serum cardiac injury biomarkers as well as cardiac lipid peroxidation, however they had evidence of an inhibition in antioxidant biomarkers. SP normalized elevated serum levels of lactate dehydrogenase (LDH), creatine kinase (CK), and CK-MB. Furthermore, SP reduced TIL-induced lipid peroxidation and oxidative stress in a dose-dependent manner.

**Conclusion:**

Administration of SP minimized the toxic effects of TIL by its free radicalscavenging and potent antioxidant activity.

## Introduction

Tilmicosin ( TIL ) is a semi-synthetic macrolide antibiotic widely used in veterinary medicine. It is a 16-member macrolide prepared by chemical modification of desmycosin. This antibiotic functions to inhibit microbial protein synthesis by binding the 50S ribosomal subunit. TIL is recommended for treatment and prevention of pneumonia associated with *Pasteurella haemolytica, Pasteurella multocida,* and *Actinobacillus pleuropneumoniae* in addition to *Staphylococcus, Streptococcus* and *Mycoplasma* species in cattle, sheep and pigs (1,3). Moreover, it is used for prevention and treatment of mastitis in ruminant animals (4,6). TIL is also used to treat respiratory infections in poultry caused by susceptible organisms (7,9). 

The efficacy of TIL is attributed to its low inhibitory concentrations (10), a large distribution volume, long elimination half-life (11,12) and other pharmacodynamic characteristics (13). However, this drug may have cardiovascular toxicity in animals. In general, overdose causes positive chronotropic and negative inotropic cardiovascular effects with increased heart rate and loss of ventricular function (14,15), particularly in young animals. This effect is mainly dependent on dose, animal species and administration route (16,17). 

In humans, accidental injection of TIL can result in chest pain, alterations in an electrocardiogram ( ECG ) and cardiac enzyme levels, including an increase in creatine kinase ( CK ) and CK-MB (18). Previous studies have definitely shown that TIL may cause elevation of cardiac damage biomarkers and oxidative stress by reducing antioxidant enzymes or by decreasing synthesis of these enzymes in the cardiac tissue (16,19). 

Spirulina platenesis ( SP ) is a unicellular cyanobacterium that has a high nutritional value and wide range of medicinal applications. SP is a very potent, naturally occurring antioxidant and free radical scavenger. Besides the free radical scavenging and antioxidant activity, SP has been shown to exhibit anti-inﬂammatory, neuroprotective, hepatoprotective, immunomodulatory and anticancer activities (20,23). SP has been reported to ameliorate organ toxicities induced by chemotherapeutic agents such as cisplatin, doxorubicin and cyclosporine (24,26). Increased interest in spirulina is based on the fact that it is believed to be non-toxic, bioavailable and provide significant multi-organ protection against numerous drugs and chemical-induced toxic assaults (27,30). Considerable data exist that show higher effectiveness of numerous chemotherapeutic agents, as well as a decrease in adverse effects when SP is administered concurrently with antioxidants (31,32). 

The main objective of this study was to investigate the possible protective effect of SP by evaluating serum cardiac damage biomarkerslactate dehydrogenase ( LDH ), CK, and CKMB as well as cardiac tissue malondialdehyde ( MDA ), reduced glutathione ( GSH ), superoxide dismutase ( SOD ), catalase ( CAT ) and total antioxidant capacity ( TAC ) levels in mice treated with TIL. 

## Materials and Methods

### Chemicals

Pure premium SP powder was purchased from HerbaForce, UK. Tilmicosin^®^( TIL; vial, 33 mg/ ml ) in clinical formulation was purchased from Arabcomid, Egypt. All kits were purchased from Biodiagnostics, Egypt except for LDH which was obtained from Randox Laboratories Ltd., UK and CK as well as CK-MB which were purchased from Stanbio™ ( TX, USA ). All other chemicals used in the experiment were analytical grade. 

### Animals and experimental design

An experimental *in vivo* animal study used 40 male albino mice that weighed 25-30 g. Animals were purchased from the Egyptian Organization for Biological Products and Vaccines. Mice were kept in a ventilated room under controlled laboratory conditions of normal light-dark cycle ( 12 hours light/dark ) and temperature ( 25 ± 2˚C ). Food and water were provided ad libitum. The animals were treated in accordance with the Guidelines for Animal Experimentation of the Ethics Review Committee of the Faculty of Veterinary Medicine, Suez Canal University, Ismailia, Egypt ( Approval no. 20147 ). After a one week acclimation period, mice were randomly assigned to five different groups of eight animals per group. The first group was treated with normal saline. The second group received SP ( 1000 mg/ kg, oral ) for five successive days. The third group received a single dose of TIL ( 75 mg/kg, subcutaneous ). Groups 4 and 5 were given SP at doses of 500 and 1000 mg/kg body weight for five consecutive days just before administration of TIL at the same dose and regimen used for group 3 (19). 

### Serum collection and tissue preparation

At the end of the experiment ( 24 hours after TIL administration ), blood samples were collected via direct heart puncture under light ether anaesthesia. Blood samples were allowed to clot at room temperature and centrifuged at 3000 rpm for 15 minutes after which the sera were separated and stored at -20˚C as aliquots for further biochemical analysis. 

After blood collection, mice were sacrificed by deep ether anaesthesia. The hearts were rapidly excised from each animal, trimmed of connecting tissue, and washed free of blood with a 0.9% NaCl solution and distilled water. The heart tissue was blotted over a piece of filter paper and perfused with 50 mM of sodium phosphate buffer saline ( 100 mM Na_2_HPO_4_/ NaH_2_PO_4_; pH=7.4 ) in an ice cold medium that contained 0.1 mM ethylene diamine tetra acetic acid ( EDTA ) to remove any red blood cells and clots. Then, heart tissues were homogenized in 5-10 ml cold buffer per gram of tissue and centrifuged at 5000 rpm for 30 minutes. The resultant supernatant was transferred into Eppendorf tubes and preserved at -80˚C in a deep freezer until use for various biochemical assays. 

### Serum biochemical analysis

The sera stored at -20˚C were used for estimation of serum cardiac enzymes LDH, CK and CK-MB. Serum LDH activity was determined enzymatically according to the manufacturer’s protocol using kits from Randox Laboratories Ltd, UK (33). CK activity was estimated according to the method developed by Szasz et al. (34) using a Stanbio™ CK-NAC ( UV-Rate ) Kit ( TX, USA ). Serum CK-MB activity was assessed by an immunoinhibition method developed by Wurzburg et al. (35) using a Stanbio™ Creatine Kinase MB Kit ( TX, USA ). 

### Evaluation of tissue lipid peroxidation and antioxidant enzymes

Lipid peroxidation was evaluated by measurement of MDA content in cardiac tissues according to Mihara and Uchiyama (36). The non-enzymatic antioxidant biomarker, GSH was assessed according to Beutler et al. (37). The enzymatic antioxidant biomarkers, SOD was evaluated according to Nishikimi et al. (38) and CAT according to Aebi (39). In addition, TAC was determined according to Koracevic et al. (40). 

### Statistical analysis

Data are presented as mean ± standard error of mean ( SEM ). Statistical significance was assessed by the statistical package for social sciences ( SPSS ) version 16. For comparison, we used the one-way analysis of variance ( ANOVA ) test and a post-comparison was carried out with Duncan’s multiple range test for post hoc analysis. Statistical significance was acceptable at the level of p≤0.05. 

## Results

### Serum biochemical analysis

The effects of TIL intoxication as well as the preventive effects of SP on serum biochemical analysis are shown in table 1. There were significant increases ( p≤0.05 ) in serum cardiac injury markers LDH ( 213.88% ), CK ( 390.16% ) and CK-MB ( 900.64% ) recorded in TIL intoxicated rats compared to the untreated control group. 

Pre-treatment with SP at doses of 500 and 1000 mg/kg for 5 consecutive days prior to TIL intoxication dose-dependently ameliorated the changes in the majority of studied serum parameters. The results indicated that SP effectively reduced TILinduced cardiotoxicity. SP pre-administration at a dose of 500 mg/kg significantly ( p≤0.05 ) reduced serum cardiac injury biomarkers LDH ( approximately 63.73% ), CK ( 43.48% ) and CK-MB ( 56.37% ). At a dose of 1000 mg/kg, LDH ( approximately 56.16% ), CK ( 28.28% ) and CK-MB ( 28.38% ) biomarkers showed significant ( p≤0.05 ) reductions. 

There were no significant changes in serum biomarkers in rats that received only SP at a dose of 1000 mg/kg ( group 2 ) when compared to the control group, which indicated the safety of SP at the selected doses used in this study. 

### Cardiac lipid peroxidation and antioxidant biomarkers

The effects of TIL intoxication as well as preventive effects of SP on cardiac tissue homogenate lipid peroxidation and antioxidant parameters are shown in table 2. A significant increase ( p≤0.05 ) in cardiac MDA content ( 234.20% ) was observed compared to the control group. On the other hand, cardiac GSH ( 31.66% ), SOD ( 48.33% ), CAT ( 30.25% ) and TAC ( 82.88% ) significantly ( p≤0.05 ) decreased. Concerning the TIL-SP500 group, cardiac MDA levels decreased ( 52.46% ) whereas increases were observed for GSH ( 240.62% ), SOD ( 146.48% ), CAT ( 164.62% ), and TAC ( 112.56% ) compared to the TIL-intoxicated group. 

With regards to the TIL-SP1000 group, cardiac MDA decreased 39.15%, while increases were observed in GHS ( 307.17% ), SOD ( 183.91% ), CAT ( 310.66% ), and TAC ( 121.13% ). 

**Table 1:** Serum enzyme activities and biochemical parameters in control and treated groups


	LDH (U/L)	CK (U/L)	CK-MB (U/L)

**Control**	562.49 ± 25.91	122.62 ± 6.72	34.42 ± 2.21
**SP1000**	506.15 ± 21.67	118.09 ± 4.05	34.39 ± 1.81
**TIL**	1202.29 * ± 30.59	476.71 * ± 17.90	310.01 * ± 11.47
**TIL SP500**	766.38 ** ± 19.94	207.77 ** ± 16.81	174.75 ** ± 7.97
**TIL SP1000**	675.88 ** ± 16.14	134.83 ** ± 9.42	88.01 ** ± 6.54


Data are expressed as means ± SEM (n=8). TIL; Tilmicosin, SP; *Spirulina platensis*, LDH; Lactate dehydrogenase, CK; Creatine kinase, CK-MB; Creatine kinase-MB, SEM; Standard error of the mean, *; Significantly different from normal non-treated control group (p≤0.05) and **; Significantly different from TIL-intoxicated group (p≤0.05).

**Table 2:** Cardiac oxidative stress markers and antioxidant parameters in control and treated groups


	MDA (nmol/gm)	GSH (mg/g)	SOD (u/g)	CAT (u/g)	TAC (mmol/g)

**Control**	53.89 ± 3.24	23.21 ± 0.95	53.20 ± 2.19	2.05 ± 0.18	31.68 ± 1.48
**SP1000**	44.10 ± 3.87	24.21 ± 1.09	54.53 ± 2.61	2.18 ± 0.09	33.81 ± 0.52
**TIL**	126.25^*^ ± 4.72	7.35^*^ ± 0.44	25.71^*^ ± 1.36	0.62^*^ ± 0.05	26.258^*^ ± 1.20
**TIL SP500**	66.23^**^ ± 4.20	17.69^**^ ± 1.28	37.67^**^ ± 1.43	1.02^**^ ± 0.06	29.56^**^ ± 1.18
**TIL SP1000**	49.42^**^ ± 3.18	22.58^**^ ± 1.01	47.28^**^ ± 1.48	1.92^**^ ± 0.15	31.81^**^ ± 0.52


Data are expressed as means ± SEM (n=8). TIL; Tilmicosin, SP; Spirulina platensis, MDA; Malondialdehyde, GSH; Reduced glutathione, SOD; Superoxide dismutase, CAT; Catalase, TAC; Total antioxidant capacity, SEM; Standard error of the mean, *; Significantly different from normal non-treated control group (p≤0.05) and **; Significantly different from TIL-intoxicated group (p≤0.05).

## Discussion

Reactive oxygen species (ROS) are continuously generated inside an animal’s body as a consequence of exposure to numerous exogenous drugs and xenobiotics in our environment and/or many endogenous metabolic processes that involve redox enzymes and the bioenergetic electron transport mechanism (41, 42). Under normal conditions, the ROS generated are neutralized by endogenous antioxidants. A balance between the generated ROS and antioxidants exist (41). Harmful effects caused by ROS occur as a result of ROS over-production leading to disturbances in cell physiology and different pathological states (41, 42).

The ROS readily induce oxidative damage to various biomolecules including proteins, lipids, lipoproteins and DNA (42, 43). This oxidative damage has been considered an important etiological factor implicated in several chronic diseases such as neurodegenerative diseases, atherosclerosis, diabetes mellitus, arthritis, and cancer (42, 44-47). Oxidative stress affects many cellular functions through various mechanisms such as induction of mitochondrial permeability with lethal consequences or alteration in gene expression by activation of the transcription factor; nuclear factor-κB (NF-κB) (48).

Drug-induced cardiotoxicity is considered a major limitation in standard and high-dose macrolide antibiotics, a fact which is particularity applicable for TIL treatment (14,15). Oxidative stress plays a major role in TIL-induced cardiotoxicity during the normal clinical regimens of treatment which results in dose escalation limitation and hindrance of the clinical outcome as a consequence (16,19). 

As a result of TIL-induced oxidative stress, cardiac side effects are seen either when given alone or in combination with other cardiotoxic agents (49). 

Although several studies of TIL toxicity have been published, few were performed regarding the use of natural products for prevention of this toxicity and different mechanisms of their ameliorative actions. 

In the present study, cardiotoxicity caused by TIL might be attributed to the oxidative stress that resulted from free radical production. TIL intoxication increased serum cardiac injury biomarkers LDH, CK and CK-MB. Lipid peroxidation increased through elevated cardiac MDA levels, decreased cardiac enzymes SOD and CAT, as well as non-enzymatic GSH antioxidant levels (Tables1, 2). In addition, TAC was reduced by the TILintoxicated group compared to the normal control group. 

All these effects are involved in the cascade of events leading to TIL-mediated cardiac oxidative stress and toxicity. This indicates that cardiac injuries induced by TIL result from oxidative stress that arises as a result of excessive generation of ROS, which have been reported to attack various biological molecules such as lipids and cause lipid peroxidation. The activities of antioxidant enzymes, including the enzymes involved in glutathione metabolism were also perturbed in the TIL treated group (Table 2) which showed involvement of oxidative stress in TIL-mediated cardiac injury. These results were consistent with the literature (16,19) and point towards the role of ROS in TIL-mediated injury and toxicity. 

TIL toxicity appears to affect the cardiovascular system (14,19). The clinical evidence of this toxicity is generally a manifestation of the negative inotropic and positive chronotropic cardiovascular effects (19). Toxicological data indicate that lethal doses of TIL are accompanied by an increase in heart rate and an altered myocardial contractility (15). In conscious dogs, intravenous administration of TIL ( 2.5 mg/kg, body weight ) has resulted in a pronounced myocardial depression ( negative inotropy ), sinus tachycardia, and a reduction in arterial pulse pressure (15). The mechanisms of cardiotoxicity induced by macrolides, including TIL, have not been clearly suggested in the literature. 

Tamargo et al. (50) reported direct TIL cardiotoxicity due to release of epinephrine which resulted in cardiovascular overload. 

Another suggestion is that TIL’s mechanism of toxicity may be mediated through intracellular calcium. A rapid depletion of intracellular calcium through interference with sarcolemmal calcium channels may be resulted in negative inotropic effects (15). It has been reported that the macrolides josamycin and erythromycin inhibited transmembrane calcium ﬂux (50). Calcium channel blockers have been reported to have a negative inotropic effect mediated through direct antagonism of calcium ions (51). While there is the possibility that TIL acts similarly to some other macrolide antibiotics that alter intracellular calcium ﬂux, this has not been established (15). 

Additionally the toxic and potentially fatal doses of TIL appear to depend on the route of administration and the animal species. Severe toxicity has been reported in doses as low as 5 mg/kg body weight, intravenously in cattle ( death ), 30 mg/kg body weight, intramuscularly in a rhesus monkey ( death ) and 10 mg/kg body weight, intramuscularly in swine ( convulsions ) (14). Factors contributing to TIL-induced cardiotoxicity include dosage of the drug, significant pre-existing systemic illness or circulatory dysfunction, and conditions that directly or indirectly interfere with the normal physiologic responses of the heart and blood vessels (52). Multiple ventricular septal defects have been observed at necropsy of a lamb that suddenly died after a TIL injection (53). 

Unintentional human injection of TIL led to cardiac symptoms and laboratory evidence of myocardial injury (18). In mice, TIL-induced cardiotoxic effects included increased serum CK, CK-MB and total sialic acid levels as well as altering serum and cardiac tissue GSH, SOD and MDA concentrations. These alterations were enhanced 142 Spirulina by preadministration of the natural antioxidant Lcarnitine (16,19). 

In the current study, pre-administration of SP ( 500 and 1000 mg/kg ) reduced both the serum cardiac injury biomarkers and lipid peroxidation in cardiac tissues. In addition, there were dose-dependent elevations of cardiac antioxidant enzymes and glutathione levels due to SP. The antioxidant and protective effects of SP were attributed to their active antioxidant constituents such as C-phycocyanins, β carotene, vitamins, minerals, proteins, lipids and carbohydrates (54). Numerous studies have shown the cardioprotective effects of SP and its active constituents against drugs, chemicals and xenobiotic-induced cardiopathy (26,30,54). SP has a hepatonephroprotective effect against deltamethrin-induced hepatonephrotoxicity (55). The hepatoprotective and antioxidant effects of SP have been reported against CCl_4_-induced hepatotoxicity (29,56). Nephroprotective effects of SP have been reported against gentamicin-induced renal injury (57,58). Moreover, SP antioxidant effects were reported against sodium ﬂuoride-induced oxidative alterations in the offspring of pregnant rats (59). 

In another study, SP fed to pregnant rats alleviated lead-induced brain damage in newborns (60). 

Pre-treatment with SP might reduce the toxic effect of cadmium; its antioxidant properties seemed to mediate a protective effect as indicated by reductions of MDA and nitric oxide ( NO ) as well as elevations of GSH and SOD levels in liver tissue (61). In another gentamicin-induced renal injury study, 1000 mg/kg SP ( similar to the dose used in our study ) elicited significant protective activity by decreasing urea, creatinine, MDA and NO levels and elevating SOD, GSH, and GSH-Px levels, which indicated the therapeutic potential of SP against gentamicin-induced nephrotoxicity and oxidative stress (58). 

The protective effect of SP against TIL-induced oxidative stress in our study could be either direct by inhibiting lipid peroxidation and scavenging free radicals or indirect through the enhancement of the activities of SOD and CAT, enzyme-free radical scavengers in the cells. These properties could be attributed to the high levels of antioxidants such as c-phyocyanin, carotenoids, minerals and vitamins reported in SP (54). Therefore, SP could be used to prevent and treat cardiac diseases, especially those induced by oxidative damage. 

In the present study, SP administered alone at the higher selected dose of 1000 mg/kg induced nonsignificant changes in all studied serum and cardiac biochemical parameters when compared with the control group. These results were concurrent with our earlier study which used the same dose for male Wister rats, which was proven to be safe (55). 

Ishimi et al. (62) proved the safety of SP for both rats and mice. In 2011, the Dietary Supplements Information Expert Committee ( DSI-EC ) undertook a safety evaluation of SP. DSI-EC reviewed information from animal studies, human clinical trials, and pharmacopeial and regulatory sources. They analyzed 31 adverse event reports on spirulina to evaluate potential health concerns. At the conclusion of this review, DSI-EC assigned a Class A safety rating to SP, thereby permitting the admission of quality monographs for these dietary supplement ingredients in the United States Pharmacopeia and National Formulary ( USP-NF ) (63). 

## Conclusion

Drug-induced cardiotoxicity remains a major limitation in standard and high-dose macrolide antibiotics, a fact which is particularity applicable for TIL treatment. Oxidative stress plays a major role in TIL-induced cardiotoxicity during the normal clinical regimens of treatment, which results in limitations to dose escalations and hindrance of the clinical outcome as a consequence. Antioxidants have proven to be effective in ameliorating macrolide-induced toxicity in many previous studies. SP is a potent antioxidant which is reported to have cardioprotective effects, enhance the effects of many known chemotherapeutic agents, and also reduce their toxicities. 

In the present study, TIL administration clearly resulted in varying degrees of lipid peroxidation, inhibition in antioxidant enzymes’ activities and alterations in serum biochemical parameters of mice. SP treatment prior to TIL provided near complete protection in terms of serum biochemical changes, cardiac antioxidant biomarker activity and oxidativestress. 
